# Regarding: ‘Clinical value of preoperative serum calcium level in predicting deep infiltrative endometriosis among ovarian endometrioma’

**DOI:** 10.1080/07853890.2025.2596541

**Published:** 2025-12-02

**Authors:** Huan Shi, Hongkai Shang

**Affiliations:** ^a^ The Fourth Clinical School of Medicine, Zhejiang Chinese Medical University, Hangzhou First People’s Hospital, Hangzhou, China; ^b^ Department of Gynecology, Affiliated Hangzhou First People’s Hospital, School of Medicine, Westlake University, Hangzhou, China

Dear Editor

We read with interest of the study by Jiang et al. which reports that albumin-adjusted calcium (adj-Ca) is lower in women with endometriosis and is independently associated with deep infiltrating endometriosis (DIE), and that adding adj-Ca to CA-125 slightly improves discrimination (area under the curve [AUC], 0.810 vs 0.806). We commend the authors for their effort to leverage inexpensive, routinely available laboratory tests to aid surgical planning [1]. However, the choice of analytes and the control of pre-analytical factors may have a substantial impact on the study findings and their clinical interpretation.

First, it is essential to select the calcium metric that best reflects the underlying biology. Ionized calcium (iCa) is the physiologically active fraction and is pH-dependent. Recommendations from the International Federation of Clinical Chemistry and Laboratory Medicine (IFCC) advise anaerobic sampling, prompt analysis, concomitant pH measurement, and minimal heparin use to avoid spuriously low iCa. When iCa is unavailable, clinicians often rely on albumin-adjusted calcium; however, recent large-scale evidence indicates that, in most clinical settings, unadjusted total calcium is the most accurate and practical surrogate for iCa [2]. Albumin-adjusted calcium can be substantially biased in cases of hypoalbuminemia, renal dysfunction, and acid-base balance disorders. We therefore recommend including unadjusted total calcium as the appropriate calcium index in sensitivity analyses. Moreover, because adj-Ca is computed from total calcium and albumin, model specifications should avoid including both albumin and adj-Ca simultaneously to prevent mathematical coupling and collinearity.

Second, the incremental gain observed when adding adj-Ca to CA-125 appears small. A formal DeLong test for correlated receiver operating characteristic (ROC) curves should be reported, along with net reclassification improvement (NRI) and decision curve analysis (DCA), to quantify whether adj-Ca meaningfully changes classification or net clinical benefit across plausible threshold probabilities. Bootstrap internal validation could also be used to assess any optimism in model performance. Without such analyses, it is difficult to infer actionable value from a 0.004 increase in AUC [3].

Third, serum calcium is influenced by parathyroid hormone, 25-hydroxyvitamin D, magnesium, phosphate, kidney function (eGFR), and commonly used medications (e.g. proton-pump inhibitors *via* hypomagnesemia, thiazide diuretics). If these data are available, we recommend reporting these variables and performing restricted and sensitivity analyses [4].

In summary, the concept exhibits significant translational potential. The successful adoption of these findings in clinical settings will ultimately be determined by the appropriate calcium metric, avoiding collinearity, demonstrating clinically meaningful incremental value, and tightening control of confounding and preanalytical factors.

Sincerely, Hongkai Shang

## Data Availability

No new data were generated during the study.
